# Effect of Esomeprazole Treatment on Extracellular Tumor pH in a Preclinical Model of Prostate Cancer by MRI-CEST Tumor pH Imaging

**DOI:** 10.3390/metabo13010048

**Published:** 2022-12-28

**Authors:** Pietro Irrera, Miriam Roberto, Lorena Consolino, Annasofia Anemone, Daisy Villano, Victor Navarro-Tableros, Antonella Carella, Walter Dastrù, Silvio Aime, Dario Livio Longo

**Affiliations:** 1 Department of Environmental Biological and Pharmaceutical Sciences and Technologies, University of Campania “Luigi Vanvitelli”, 81100 Caserta, Italy; 2 Institute of Biostructures and Bioimaging (IBB), National Research Council of Italy (CNR), 10126 Turin, Italy; 3 Department of Molecular Biotechnology and Health Sciences, University of Turin, 10126 Turin, Italy; 4 Department of Nanomedicines and Theranostics, Institute for Experimental Molecular Imaging, RWTH Aachen University, 52074 Aachen, Germany; 5 IRCCS SDN SynLab, 80143 Naples, Italy

**Keywords:** tumor, cancer, prostate cancer, magnetic resonance imaging (MRI), chemical exchange saturation transfer (CEST), iopamidol, pH, acidosis, treatment, proton pump inhibitors (PPIs), resistance

## Abstract

Novel anticancer treatments target the pH regulating system that plays a major role in tumor progression by creating an acidic microenvironment, although few studies have addressed their effect on tumor acidosis. In this study, we investigated in vivo several proton pump inhibitors (PPIs) targeting NHE-1 (Amiloride and Cariporide) and V-ATPase (Esomeprazole and Lansoprazole) proton transporters in the DU145 androgen-insensitive human prostate cancer model. In cellulo results showed that DU145 are sensitive, with decreasing efficacy, to Amiloride, Esomeprazole and Lansoprazole, with marked cell toxicity both in normoxia and in hypoxia, with almost any change in pH. In vivo studies were performed upon administration of Esomeprazole to assess both the acute and chronic effects, and Iopamidol-based tumor pH imaging was performed to evaluate tumor acidosis. Although statistically significant tumor pH changes were observed a few hours after Esomeprazole administration in both the acute study and up to one week of treatment in the chronic study, longer treatment resulted in a lack of changes in tumor acidosis, which was associated to similar tumor growth curves between treated and control groups in both the subcutaneous and orthotopic models. Overall, this study highlights MRI-CEST tumor pH imaging as a valid approach to monitoring treatment response to PPIs.

## 1. Introduction

Prostate cancer is one of the three major responsible causes of death in men diagnosed with cancer [1,2], and in recent decades, different approaches, from imaging to screening assays, have been combined to help clinicians to better stratify the progression risks and characterize tumor lesions in a patient-based fashion [3,4]. Above all, multi-parametric Magnetic Resonance Imaging (MRI) is one of the preferred imaging techniques able to provide both anatomical and functional information about perfusion, oxygenation and necrosis induced by the radiotherapy [5]. Thus, many efforts have been focused on the standardization and validation of the multiple approaches available in clinic [6,7]. Nevertheless, other assays are needed to complete the screening, such as biopsies and prostate-specific antigen (PSA) tests that look into specific biomarkers for achieving the best prognosis for patients [8]. An emerging MRI approach is the Chemical Exchange Saturation Transfer (CEST) imaging technique, which by exploiting pH sensitive agents can provide measurements of tissue pH [9,10,11]. In particular, the administration of Iopamidol, a pH-responsive contrast agent, allows accurate quantitative assessments of extracellular tumor pH in vivo [9,12,13,14]. Of note, some studies have been conducted in human patients with promising clinical translatability for the characterization of tumor lesions with accurate pH measurements [15,16,17]. It is well known that the tumor microenvironment is characterized by acidosis, a physiological state that is exacerbated by the abnormal metabolism rate that tumors adopt to survive and to counteract the defensive mechanism of the organism [18,19,20,21], especially to evade and suppress the immune surveillance [22,23]. As a matter of fact, immune cells suffer this condition and become harmless to the tumor cells since they are no longer able to fight against the neoplastic cells [24,25]. Consequently, the tumor cells can grow, advance in more aggressive stages, and finally migrate from the primary mass to the circulation, creating metastasis in multiple target organs, with tumor acidosis being associated to cancer invasiveness [26,27,28,29,30]. Therefore, treatments able to perturb this acidic condition have been extensively investigated [31]. In particular, alkalinization therapies showed promising results in reducing cancer invasiveness, by strengthening the immune activation when coupled with immunotherapy [32,33,34] and by enhancing cytotoxicity when coupled with pharmaceuticals [35]. Another way to modify the extracellular pH in tumors is to actively inhibit the enzymatic pathways involved in the altered metabolism and physiology. Recently, MRI-CEST tumor pH imaging has been shown to assess the treatment response of drugs targeting different metabolic pathways by measuring tumor pH changes [36,37,38,39]. Among the several proton transporters, V-ATPases are the main transporters responsible for the efflux of protons to the extracellular milieu, and they have been found to be more active and more expressed in several types of cancer and associated with the invasiveness in prostate cancers [40]. Proton pump inhibitors (PPIs) are weak bases that upon protonation accumulate selectively in acidic spaces, as in tumors, where they are activated, and are non-toxic to normal cells [41]. These compounds block the transport of the H^+^ excess from the cytosol compartment to the extracellular space, therefore allowing to reduce tumor acidity and aggressiveness [42,43]. Promising results have been obtained in several murine models of gastric, colorectal, breast and melanoma cancers upon treatment with PPIs [41,44,45,46,47,48,49]. Therefore, our aim was to investigate the efficacy of several PPIs on a castration-resistant human prostate cancer cell line by combining in vitro and in vivo studies addressing tumor acidosis. Esomeprazole, Lansoprazole (vacuolar-ATPase inhibitors), Amiloride and Cariporide (sodium-hydrogen exchanger-1, NHE-1 inhibitors) were assayed for cell viability and extracellular medium pH measurements to monitor the therapeutic effect on tumor cells incubated for 24 or 48 h with these drugs, both in normoxia and hypoxia. Tumor growth and MRI-CEST extracellular tumor pH measurements were performed in vivo after acute or chronic Esomeprazole administration in both subcutaneous and orthotopic murine models for monitoring the treatment outcome.

## 2. Materials and Methods

### 2.1. Cell Culture

The DU145 cell line was obtained from American Tissue Culture Collection (ATCC) (Manassas, VA, USA). DU145 cells were grown in Minimal Essential Medium (MEM) with the addition of 1% (*v*/*v*) non-essential amino acids (NEAA), 10 % (*v*/*v*) fetal bovine serum (FBS), 1% (*v*/*v*) Na-Pyruvate, 100 mg/mL streptomycin and 100 U/mL penicillin, bought from Lonza (Lonza Sales AG, Verviers, Belgium). Cells were incubated inside 175 cm^2^ flasks at 37 °C in a humidified 5% CO_2_ incubator, and after reaching confluence, detaching was obtained with the addition of 2 mL of Trypsin–EDTA solution.

### 2.2. In Cellulo Treatment with PPIs

Esomeprazole, Lansoprazole, Amiloride and Cariporide were obtained from Sigma (Sigma Aldrich, Milano, Italy). The inhibitors came as a powder and were then dissolved in DMSO to prepare a mother stock solution that was diluted at the moment of the experiment at several concentrations. A final volume of 100 µL was prepared for each well of the 96-well plate, where cells were seeded the day before at a density of 3 × 10^4^. Cell viability tests were conducted after 24 h and 48 h of drug treatments performed in normoxia and in hypoxia, whereas for pH measurements they were conducted only in normoxia after 24 h exposure. The medium of control cells was added with DMSO to match the same concentration in drug-treated cells. Cells were incubated in hypoxia with a hypoxic incubator (New Brunswick™ Galaxy^®^ 48 R, Eppendorf S.r.l., Milan, Italy) set to 1% O_2_, 5% CO_2_, and 95% humidity during the whole experiment.

### 2.3. Cell Viability Study

A colorimetric assay with the 3-(4,5-dimethylthiazol-2-yl)-2,5-diphenyltetrazolium bromide (MTT) dye was used for cell viability studies in a multi-well plate. Each well was washed twice with phosphate buffer solution (PBS) and then filled with the MTT solution (prepared by dissolving MTT in PBS) and incubated for 4 hours (in normoxic or hypoxic conditions). During the incubation, formazan crystals (purple colored) are produced following the enzymatic reaction and are dissolved in DMSO before the fluorescence reading at 500–600 nm.

### 2.4. Extracellular pH Measurements in Cellulo

Extracellular pH measurements were performed with the pH-Xtra Glycolysis Assay (Luxcel Bioscience, Cork, Ireland) kit in normoxic conditions using a microplate reader (BioTek Instruments, Inc., Winooski, VT, USA). Following the 24 h treatment in a standard incubator (5% CO_2_, 37 °C), 96-well plates were placed in a CO_2_-free incubator at 37 °C for 2 h. A detailed description of the whole procedure has been previously described [50]. The pH accuracy in calculating the extracellular pH medium is reported in Supplementary Figure S1.

### 2.5. QRT-PCR and Western Blot

Total RNA (500 ng) was extracted from Du145 prostate cancer cells by using TRIzol^®^ reagent (15596018, Invitrogen, Waltham, MA, USA) and used to obtain cDNA using the SuperScript III Reverse Transcriptase Kit (Invitrogen). The quantitative RT-PCR was carried out on a Fast Real-Time PCR System (Applied Biosystems, 7900HT instrument) using Sybr Green 2× PCR Master Mix with GAPDH as house-keeping gene, using the ΔΔCt method.

A RIPA Lysis buffer (Merk Millipore #20-188) supplemented with Protease Inhibitor Cocktail (Sigma #P2714) was used to extract proteins from the DU145 prostate cancer cells. Then, 50 mg of total protein was separated by Bio-Rad Mini-PROTEAN^®^ TGX ^TM^ Gel (Bio-rad #456-9034) and transferred to a 45 μm-pore polyvinylidene difluoride (PVDF) membrane (Immobilon PSQ, Millipore) and TBS-T (Tris-buffered saline with 0.1% Tween-20), with 5% milk used for blocking. Primary antibodies for ATP6V1A (1:1000; Abcam #137574) and NHE1 (1:2000; Novus Biological #NBP1-76847) were detected by anti-rabbit IgG (1:5000; Sigma # A6154), and β-actin (1:3000; Sigma-Aldrich #A1978) by anti-mouse IgG (1:5000; Sigma #A4416). Signals were detected with Pierce TM ECL Western Blotting Substrate kit (Thermo-Fisher #32106), and ImageJ software (https://imagej.nih.gov/ij/index.html) was used to quantify the bands.

### 2.6. Experimental In Vivo Settings

The European guidelines (directive 2010/63) were followed for animal procedures and husbandry and according to the Ethical Committee of our university. Athymic nude mice were obtained from Envigo Srl (San Pietro al Natisone, Italy) and housed in a temperature-controlled room with a 12-hour light/dark schedule. DU145 cells (5 × 10^6^ cells) were injected subcutaneously in both flanks of 8-week-old male mice in two cohorts of mice; the first was used for the acute effect experiment (*n* = 8) and the second one was used for the 3-week-long chronic regimen (*n* = 16). A third cohort of mice (*n* = 8) was used for the orthotopic model in which 1 × 10^6^ DU145 cells were inoculated into the prostate frontal lobe. For the subcutaneous model, caliper measurements were used to record the two dimensions of height (H) and length (L) to calculate tumor volumes using the formula $V=\frac{H\;x\;L^{2}}{2}$. Instead, for the orthotopic model, region of interests were placed on T_2_-weighted axial MR images by using the ITK-Snap software (version 3.6, itksnap.org). Tumors with a size of ca. 4–5 mm in diameter started the imaging protocol with systemic anesthesia obtained by intramuscle injection of a mixture of xylazine 5 mg/kg (Rompum; Bayer, Milan, Italy) and tiletamine/zolazepam 20 mg/kg (Zoletil 100; Virbac, Milan, Italy). Esomeprazole was prepared at a concentration of 1 mg/mL in a saline solution (NaCl 0.9%) and administered orally (dose = 2.5 mg/kg b.w., ca. 100 µL of volume per mouse) every other day, starting when tumor dimensions were ca. 50–80 mm^3^ (for both subcutaneous and orthotopic tumor models).

### 2.7. MRI In Vivo Experiments

A Bruker Avance Neo MRI microimaging scanner operating at 7 Tesla (Bruker Biospin, Ettlingen, Germany) and equipped with a quadrature 1H coil was used to acquire all the images. Mice were anesthetized for the MRI acquisitions, and before their placement in the scanner a tail vein catheter was placed to inject Iopamidol. The imaging protocol involves the acquisition of scout images and of a T_2_-weighted multislice sequence, followed by the Z-spectra (CEST) acquisition. CEST acquisition parameters were: B_1_ = 3 µT, TS_1_ = 3 s, TS_2_ = 1 s, offsets = 46, offset range: −10 to +10 ppm, matrix = 128 × 128, FOV = 30 × 30 mm, number of slices = 8, slice thickness = 1.5 mm, TR = 11.2 s, and TE = 3.9 ms [51]. Iopamidol was injected after the first CEST acquisition (dose: 4 g Iodine/kg b.w.) and a second CEST image was acquired to calculate the tumor extracellular pH maps. MRI-CEST pH images were acquired in the first cohort of mice (acute effect) 3 hours after the Esomeprazole administration, whereas in the second cohort of mice (chronically treated) they were acquired after the first and the second week of treatment.

### 2.8. CEST Imaging Analysis

An in-house script was used to analyze CEST images within the MATLAB (The Mathworks, Inc., Natick, MA, USA) environment. Briefly, for each voxel, Z-spectra were interpolated and B_0_-shift corrected by cubic smoothing splines and CEST contrast (ST%) was calculated by asymmetry analysis [52]. Given that the endogenous components can contribute to the CEST signal, a background subtraction was performed by subtracting the ST contrast after Iopamidol injection from the ST contrast before the injection on a per voxel basis to obtain the difference contrast map (ΔST%). Tumor pHe values were calculated in vivo by applying the ratiometric procedure [12].

### 2.9. Statistical Analysis

Data are shown in all graphs as mean values with SD (standard deviations). ANOVA analysis was applied with the post-hoc Bonferroni correction for in vitro studies, whereas an unpaired Student’s *t*-test was used for in vivo studies by using GraphPad Prism version 9.1 (La Jolla, CA, USA).

## 3. Results

### 3.1. In Cellulo Studies

Androgen-insensitive DU145 prostate cancer cells showed an elevated expression of both NHE1 and V-ATPase proton pumps at both mRNA and protein levels, comparable to that of other androgen-insensitive PC3 cancer cells (Supplementary Figure S2).

**Figure 1:**
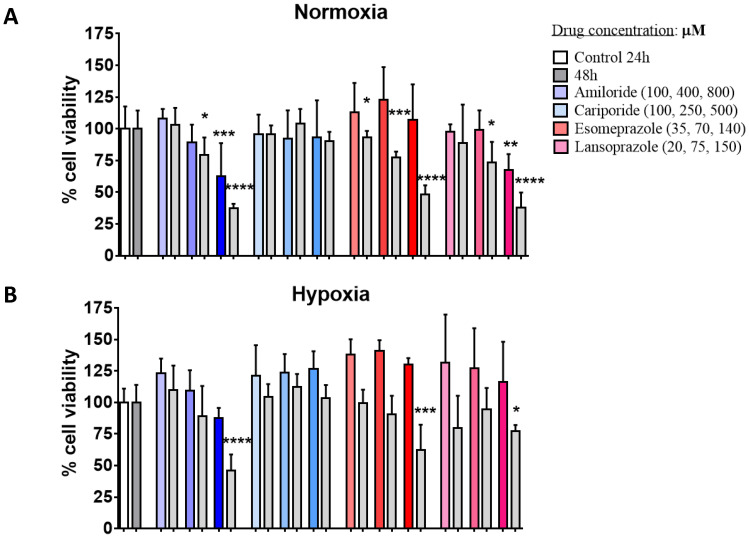
Impact of proton pump inhibitors on cell viability. Cell viability results for DU145 human prostate cancer cell line in normoxia (**A**) and hypoxia (**B**) after 24 or 48 h incubation (gray columns) with Amiloride, Cariporide, Esomeprazole, and Lansoprazole (in blue, light-blue, orange and pink bars, respectively), at increasing concentrations (associated with darker colors of the bar columns; first two columns on the left are control cells at 24h or at 48h). Statistical significance was calculated with ANOVA analysis corrected with a Bonferroni post-hoc test applied to the control group at the corresponding time point (* *p* value < 0.05; ** *p* value < 0.01; *** *p* value < 0.001; **** *p* value < 0.0001).

Amiloride was effective in reducing the cell viability of the DU145 cancer cells starting at an intermediate concentration, with higher cell death upon longer exposure (48 h) or at the highest concentration (Figure 1A). Even in hypoxic conditions, Amiloride treatment resulted in significant cell death at both 24 h and 48 h of exposure but at the highest concentration (Figure 1B). On the other hand, Cariporide was totally ineffective in decreasing cell viability at any tested condition and concentration. Esomeprazole treatment showed significant and comparable results to Cariporide, with a significant cell death at all the concentrations but only after 48 h of exposure in normoxia and at the highest concentration in hypoxic conditions. The homologue Lansoprazole induced a significant response only after incubation in normoxic and hypoxic conditions at the highest doses.

**Figure 2:**
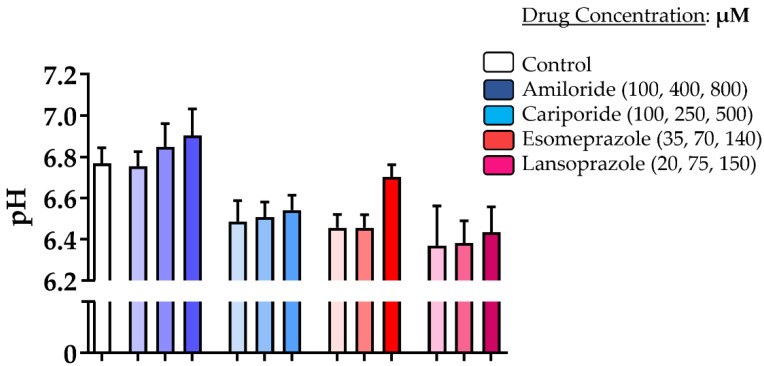
Impact of proton pump inhibitors on extracellular pH. Measurement of culture media pH values for DU145 prostate cancer cells upon incubation for 24 h in normoxia with Amiloride, Cariporide, Esomeprazole, and Lansoprazole (blue, light-blue, orange and pink bars, respectively) at increasing concentrations (associated with darker colors of the bar columns) compared to control cells (white column bar on the left).

pH measurements taken for DU145 after 24 h of incubation with the different PPIs revealed that only Amiloride was able to slightly modify the extracellular pH when compared to the untreated cells, although this was not statistically significant (Figure 2). All other drugs did not provide any extracellular pH increase compared to control cells; instead, a marked acidification was observed, albeit Esomeprazole showed a more alkaline extracellular pH at the highest concentration compared to the other two concentrations.

### 3.2. Extracellular Tumor pH Evaluation upon Acute Treatment

**Figure 3:**
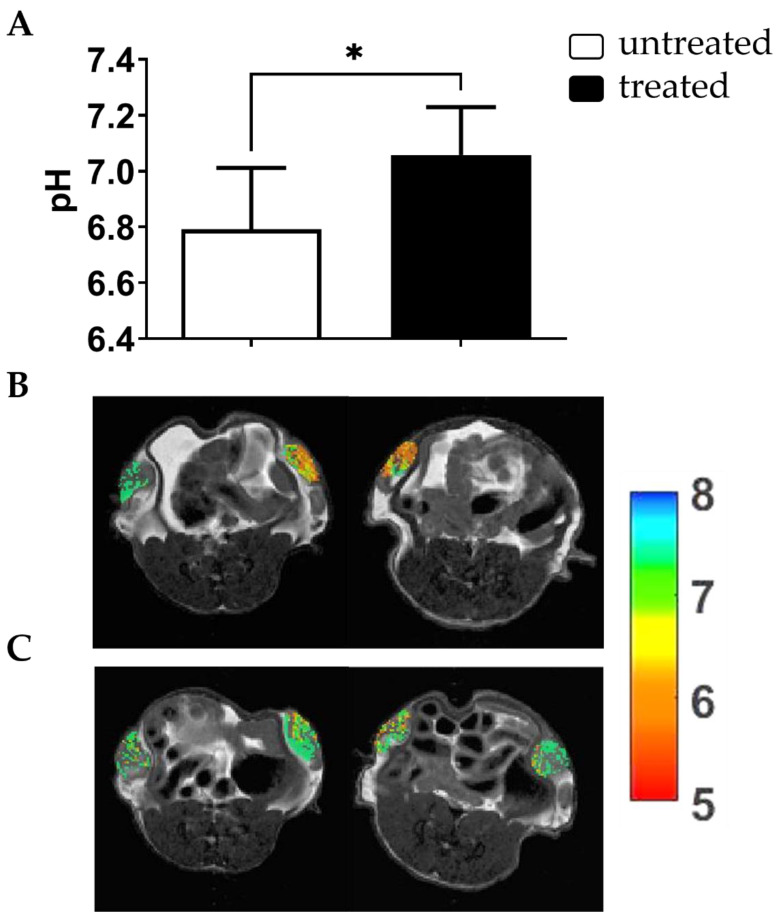
Acute effect of Esomeprazole on tumor extracellular pH. MRI-CEST extracellular tumor pH values for DU145 subcutaneous tumors three hours after oral Esomeprazole administration (dose: 2.5 mg/kg b.w.) in treated (*n* = 4) and untreated (*n* = 4) groups (* *p* value < 0.05, unpaired Student’s *t*-test) (**A**). Tumor pH maps superimposed over T_2w_ anatomical images are shown for two representative untreated mice (**B**) and for two treated mice (**C**) showing a marked reduction in tumor acidic values upon Esomeprazole administration.

Since Esomeprazole showed a marked toxicity in both normoxic and hypoxic conditions, it was selected for the following in vivo studies. Consequently, Esomeprazole was administered once to mice bearing subcutaneous DU145 tumors, and MRI-CEST tumor pH imaging was performed to evaluate acute extracellular tumor pH changes three hours after the single administration. Baseline tumor extracellular pH values showed a marked acidic tumor microenvironment, whereas a significant alkalinization occurred in the same mice after Esomeprazole gavage (tumor extracellular pH = 6.79 and 7.07 for baseline and treated mice, *p* < 0.05, Figure 3).

### 3.3. Tumor Extracellular pH Evaluation upon Chronic Esomeprazole Administration in the Subcutaneous Model

**Figure 4:**
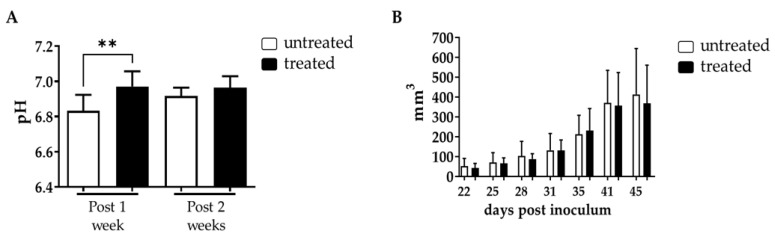
Chronic effect of Esomeprazole on tumor extracellular pH and tumor growth. Measured extracellular tumor pH values (**A**) following long-term Esomeprazole oral administration in the DU145 subcutaneous tumor murine model between untreated (*n* = 8) and treated (*n* = 8) groups after one week (28 days) and two weeks (35 days) of treatment (white and black bars, respectively, ** *p* value < 0.01). Tumor growing curves obtained from caliper measurements (**B**).

Further investigation on the effect of Esomeprazole in the DU145 subcutaneous cancer model was conducted with a prolonged treatment regimen, with mice treated every other day with Esomeprazole at 2.5 mg/kg b.w. once tumor volumes reached ca. 70 mm^3^. MRI-CEST tumor pH imaging was then performed at one and at two weeks of treatment, while caliper measurements were taken every three days to quantify tumor volume changes during the three weeks duration of the study. Tumor extracellular pH imaging showed a significant difference after one week of treatment with Esomeprazole between untreated and treated mice (tumor extracellular pH = 6.83 and 6.98 for untreated and treated mice, *p* < 0.01, Figure 4A), confirming the efficacy of Esomeprazole in altering tumor acidosis as previously observed during the acute administration study. However, two weeks after Esomeprazole administration we observed the onset of resistance, with comparable tumor pHe values between the two groups (tumor extracellular pH = 6.91 and 6.97 for untreated and treated mice, Figure 4A). Tumor growth curves showed that both untreated and treated mice had a similar growing rate until the end of the study (Figure 4B), reflecting the failure of Esomeprazole to induce tumor pH changes at longer exposure times.

**Figure 5:**
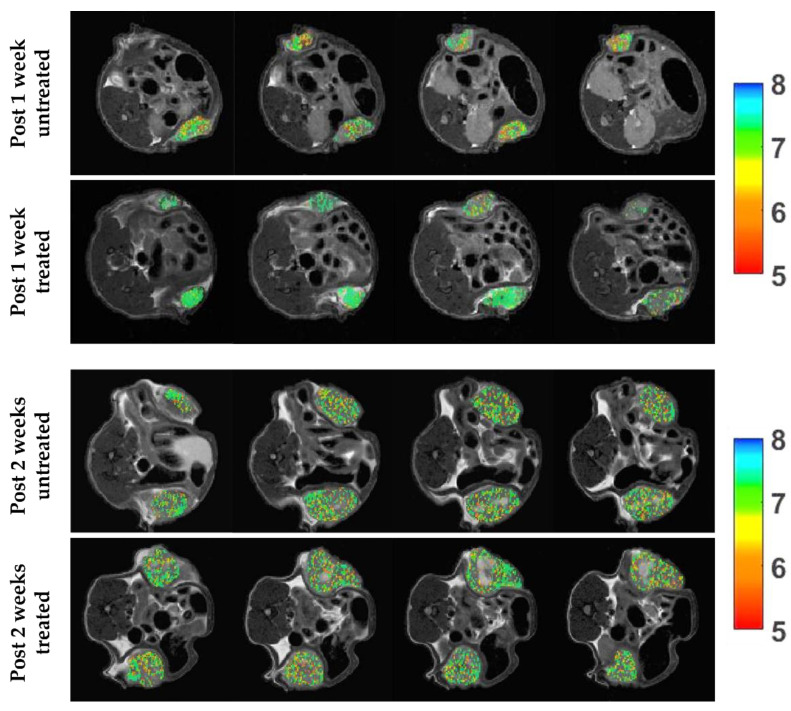
Extracellular tumor pH (pHe) images upon chronic Esomeprazole treatment. Representative tumor extracellular pH images (from top to bottom) after one week of treatment (average tumor pHe of 6.74/6.85 and of 6.98/7.05 for untreated and treated mice, respectively) and after two weeks of treatment (average tumor pHe of 6.90/6.94 and of 6.92/6.95 for untreated and treated mice, respectively).

Representative tumor extracellular pH maps for control and treated mice showed a marked reduction in tumor extracellular acidosis for Esomeprazole-treated mice after one week of treatment, in comparison to control mice, whereas extracellular tumor pH values were comparable after two weeks of treatment (Figure 5).

Analogously to the subcutaneous model, orthotopically injected mice started a two-week Esomeprazole treatment once the tumor reached an average volume of ca. 70 mm^3^, with the same dose and regimen used in the subcutaneous model. MRI-based tumor volume measurements did not show any difference in tumor growth between the two groups of mice (Supplementary Figure S3).

## 4. Discussion

The efficacy of the proton pump inhibitors Amiloride, Cariporide, Esomeprazole and Lansoprazole to alter extracellular pH was evaluated in vitro and in vivo in the human prostate cancer cell line DU145. Each inhibitor was tested in vitro at three increasing concentrations and at two exposure times of 24 and 48 h in both normoxic and hypoxic conditions. Cell viability and extracellular medium pH quantifications were performed to select the most effective inhibitor to be tested in vivo. The cell viability experiments showed that Amiloride, Esomeprazole and Lansoprazole provided a consistent decrease in cell viability with a dose-dependent trend in all the tested conditions. The toxicity in both normoxic and hypoxic conditions followed this order: Amiloride, Esomeprazole, Lansoprazole. On the other hand, Cariporide was totally ineffective in reducing cell viability at all the examined conditions. Although there were promising results regarding the efficacy of killing cancer cells, extracellular pH measurements reported only a modest alkalinization upon Amiloride treatment, whereas all the other inhibitors were unable to raise the extracellular pH above the control pH. These differences can be explained by either longer drug exposure times being needed to observe significant extracellular medium pH variations or by compensatory increased activity or expression of proton pumps being required to counteract the effect of the proton pump inhibitor. Only the highest dose of Esomeprazole provided a higher pH compared to the more acidic ones observed with the lower concentrations. Then, we evaluated the efficacy of Esomeprazole to induce tumor extracellular pH changes in vivo by exploring both an acute administration and a chronic treatment. Mice with subcutaneous DU145 tumors were treated only once and imaged for tumor pH changes three hours after the oral administration. A significant and marked increase in extracellular tumor pH was observed, indicating a clear in vivo effect in inhibiting the targeted V-ATPase proton pump transporters. Based on these results, we further tested the Esomeprazole efficacy upon a repeated administration to mice every other day for two and three weeks, in an orthotopic and subcutaneous model, respectively. MRI-CEST tumor pH imaging was performed in the subcutaneous model after the first and second week of treatment to monitor any tumor extracellular pH change during tumor progression, while tumor volume measurements continued until the third week of treatment. In line with the acute effect study, a statistically significant higher tumor extracellular pH was found in the treated mice after the first week of treatment. However, after the second week of Esomeprazole treatment, any alteration in tumor extracellular pH was observed for the treated mice, suggesting the onset of resistance to the administered drug. Notably, any tumor volume changes were detected after three weeks of treatment, confirming the tumor pH imaging findings. In addition, the orthotopic model also confirmed the inefficacy of Esomeprazole to reduce tumor progression, since any tumor volume changes were detected during the two weeks of treatment between control and treated mice. The DU145 human prostate cancer cell line showed a marked expression of both NHE1 and V-ATPase proton pumps, comparable to that observed for the androgen-insensitive PC3 human prostate cancer cell line, hence representing a promising anti-cancer target for PPIs [53,54,55]. However, one limitation of this study is that we did not evaluate their expression in tumors, since changes in their relative expression or activity upon chronic Esomeprazole treatment could potentially explain the lack of extracellular tumor pH changes after two weeks of treatment.

Overall, Esomeprazole is capable of inducing tumor extracellular pH changes in the DU145 animal model, but not in a sustained way for altering tumor progression. Nonetheless, targeting the pH regulatory systems seems to be a promising strategy to alter the tumor microenvironment, and the induced early tumor extracellular pH changes could be beneficial once an additional treatment is coupled with the pH-interfering drug [41,56,57,58]. In fact, several studies reported tumor growth reduction following PPIs treatment alone or in combination with other chemotherapies in several tumor models, such as lymphoma, breast, or colorectal cancers [47,59,60,61,62,63]. In spite of the positive results obtained in other tumor models, PPIs can have a different outcome when administered to prostate cancer models. In our previous work, Esomeprazole was effective in inducing only acute tumor pH changes in the PC3 prostate cancer murine model, whereas chronic treatment did not show any alteration in tumor extracellular pH or in tumor progression in both subcutaneous and orthotopic murine models, similar to what has been observed in this study upon long-term treatment [50]. In another study, PPIs treatment can even enhance tumor progression, as observed in the murine model of LNCaP prostate cancer [64]. Interestingly, other studies have pointed out the risk associated with prolonged PPIs treatment, with the consequent development of neuroendocrine tumors [65], hyperplastic gland polyps and a 39% larger mortality in prostate cancer patients, although further validation of the potentially negative association of PPIs with prostate cancer is still needed [66]. Consequently, PPIs administration must be carefully considered and likely tailored to specific cancer patients. From this perspective, MRI-CEST Iopamidol-based tumor pH imaging was essential to obtain insights about the therapeutic efficacy of this inhibitor. Many studies that exploited this non-invasive imaging technique for addressing important questions regarding the characterization of cancer metabolism and aggressiveness [13,28] and for monitoring therapeutic responses [36,39,67,68] confirmed the importance of tumor extracellular pH as a promising novel biomarker, although further studies are needed to clearly assess the potential clinical utility.

## 5. Conclusions

PPIs were able to perturb in vitro DU145 prostate tumor cell viability, and to a lesser extent, the extracellular acidification. Although acute and early Esomeprazole treatment induced significant changes in tumor extracellular pH values, chronic treatment did not affect either tumor acidosis or tumor growth. The proposed study demonstrates the crucial role of longitudinal in vivo tumor pH quantifications to properly evaluate the treatment response to novel anticancer therapies.

## Data Availability

The data presented in this study are available from the corresponding author on reasonable request. The data are not publicly available due to ongoing research.

## Supplementary Materials

The following supporting information can be downloaded at: https://www.mdpi.com/article/10.3390/metabo13010048/s1, Figure S1: pH accuracy in measuring extracellular medium pH; Figure S2: RT-PCR and WB expression of NHE1 and V-APTase in DU145 and PC3 prostate cancer cells; Figure S3: Tumor volume upon chronic Esomeprazole treatment for the DU145 prostate orthotopic murine model.
