# Supplementary material for: Effect of Esomeprazole Treatment on Extracellular Tumor pH in a Preclinical Model of Prostate Cancer by MRI-CEST Tumor pH Imaging
Source: Metabolites. 2022 Dec 28;13(1):48. doi: 10.3390/metabo13010048 (PMC9866131; doi:10.3390/metabo13010048)
Supplement: Supplementary file 1 [file metabolites-13-00048-s001.zip › metabolites-2112108-supplementary.pdf]

SUPPLEMENTARY MATERIAL

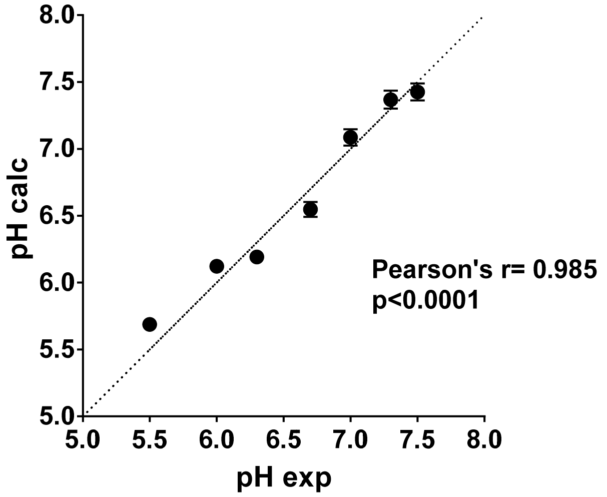

Figure S1. Calculated pH values vs experimental measured ones obtained with the pH-Xtra Glycolysis Assay.

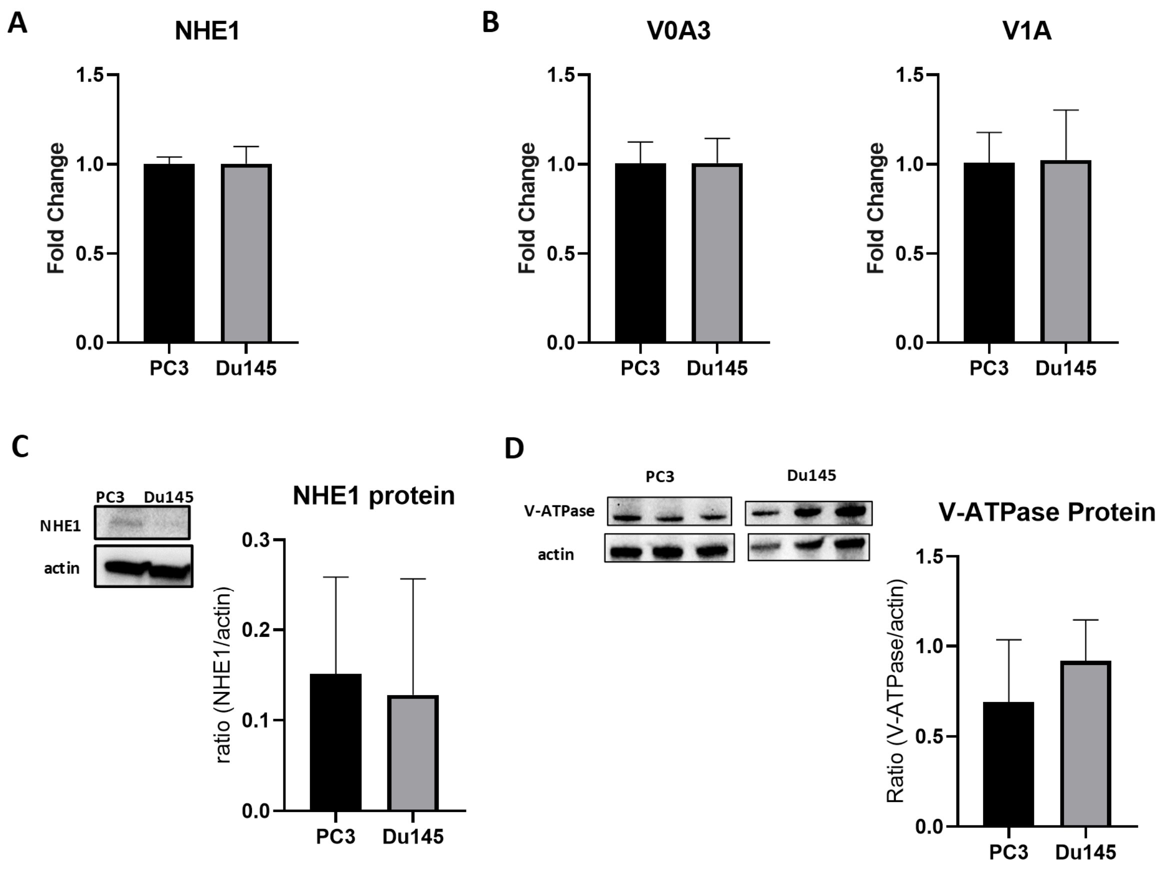

Figure S2. mRNA expression for the proton transporters NHE1 (A) and for the two subunits V0A3 and V1A for the V-ATPase (B); WB and corresponding quantification of protein levels for the NHE1 (C) and V-ATPase (D) for the two androgen-insensitive prostate DU145 and PC3 cancer cell lines.

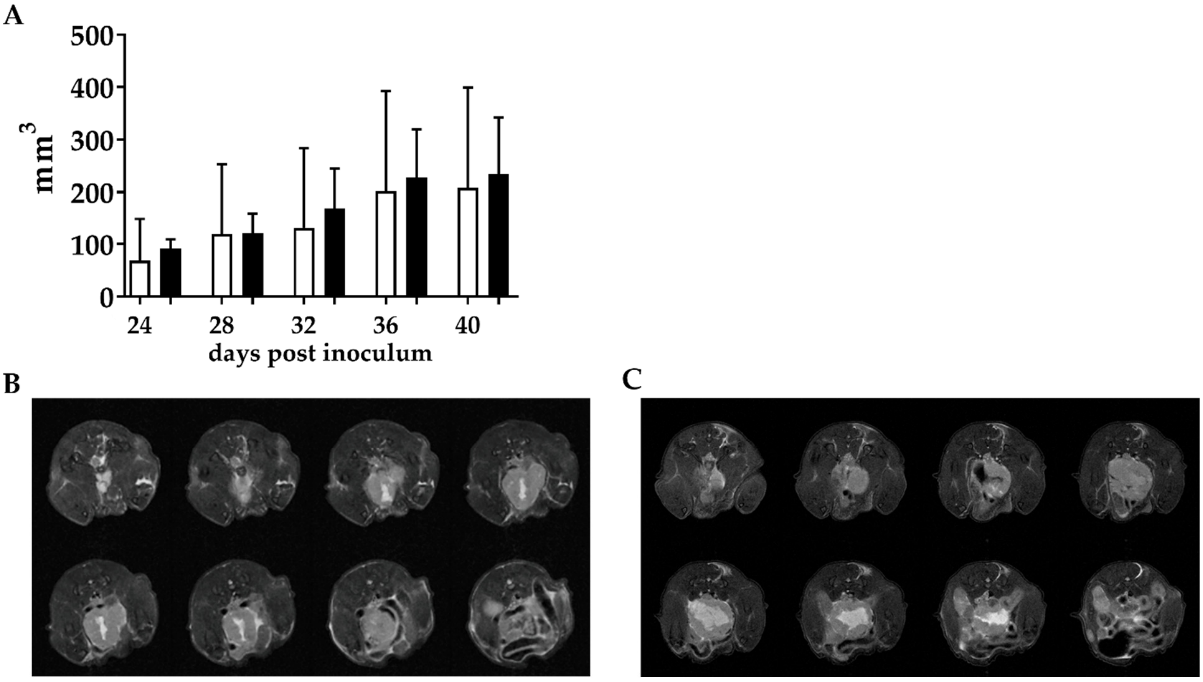

Figure S3. Tumor volume upon chronic Esomeprazole treatment for the DU145 prostate orthotopic murine model. Tumor volumes were measured from MRI T_2-weighted anatomical images for untreated (n = 4) and treated (n = 4) groups (A). Representative T_2-weighted anatomical images of orthotopic DU145 prostate tumors (8 slices) of untreated (B) and treated mice (C) at one-week post-Esomeprazole treatment.
